# Supplementary material for: Comparative genomics provides new insights into the diversity, physiology, and sexuality of the only industrially exploited tremellomycete: Phaffia rhodozyma
Source: BMC Genomics. 2016 Nov 9;17:901. doi: 10.1186/s12864-016-3244-7 (PMC5103461; doi:10.1186/s12864-016-3244-7)
Supplement: Additional file 6: — List of orphan genes with links to PFAM (related to Additional file 1: Table S1). (ZIP 1428 kb) [file 12864_2016_3244_MOESM6_ESM.zip › PFAM_UNIQUE/G04612_P.html]

#                                                                            --- full sequence --- -------------- this domain -------------   hmm coord   ali coord   env coord
  
# target name        accession   tlen query name           accession   qlen   E-value  score  bias   #  of  c-Evalue  i-Evalue  score  bias  from    to  from    to  from    to  acc description of target
  
#------------------- ---------- ----- -------------------- ---------- ----- --------- ------ ----- --- --- --------- --------- ------ ----- ----- ----- ----- ----- ----- ----- ---- ---------------------
  
G04612\_P             -            490 Ashwin               PF15323.1    215       5.9    5.5   7.0   1   1     0.019        16    4.0   7.0   133   193   259   318   217   385 0.72 - Developmental protein  
G04612\_P             -            490 ATG16                PF08614.6    194      0.69    8.4   3.5   1   1    0.0063       1.6    7.2   3.5    20   113   210   306   204   323 0.79 - Autophagy protein 16 (ATG16)  
G04612\_P             -            490 CDC27                PF09507.5    430       1.1    7.1  14.2   1   2     0.063       9.9    3.9   1.2   192   266    87   159    44   183 0.57 - DNA polymerase subunit Cdc27  
G04612\_P             -            490 CDC27                PF09507.5    430       1.1    7.1  14.2   2   2    0.0028      0.45    8.3   5.9   159   356   250   454   186   462 0.53 - DNA polymerase subunit Cdc27  
G04612\_P             -            490 Chorion\_2            PF03964.10    99      0.49    9.6   5.2   1   1    0.0013       1.3    8.3   5.2    36    63   246   281   192   316 0.65 - Chorion family 2  
G04612\_P             -            490 DUF4054              PF13262.1    106       4.5    6.2   9.3   1   1    0.0054        33    3.5   9.3    14    87   183   277   170   294 0.77 - Protein of unknown function (DUF4054)  
G04612\_P             -            490 TRAPPC-Trs85         PF12739.2    414       8.3    3.6  10.2   1   1    0.0056        11    3.2   9.8   134   213   204   290   126   312 0.74 - ER-Golgi trafficking TRAPP I complex 85 kDa subunit
